# Genotype-by-environment interactive effects and conflict solving during gonadal sex differentiation of pejerrey *Odontesthes bonariensis*, a fish with dual genotypic/environmental sex determination

**DOI:** 10.1186/s13293-025-00768-7

**Published:** 2025-10-16

**Authors:** Chenyan Wu, Wakaba Baba, Ryuichi Nakagawa, Yoji Yamamoto, Carlos Augusto Strüssmann

**Affiliations:** Department of Marine Biosciences, Graduate School of Marine Science and Technology, Tokyo University of Marine Science and Technology, Minato 4-5-7, Tokyo, 108-8477 Japan

**Keywords:** Genotypic sex determination, Environmental sex determination, Temperature-dependent sex determination, Critical period of sex determination, Genotype-environment interaction, Sex ratios, Climate change, Fish, Global warming

## Abstract

**Background:**

Genotypic (GSD) and environmental (ESD) sex determination coexist in many species of reptiles, fish, and amphibians. Inherited genotypic signals and environmental factors conceivably interact as pro-testis or pro-ovary signals during sex determination, but how such interactions affect gonadal sex differentiation in these species remains largely unexplained. This study uses a model gonochoristic fish with coexisting GSD and ESD, the pejerrey *Odontesthes bonariensis*, to examine how synergism and antagonism between sex genotype (XX/XY) and thermal (feminizing/masculinizing) regimes interactively affect environmental sensitiveness and the critical time of environmental sex determination as well as how genotype-by-environment conflicts are resolved.

**Methods:**

We performed a series of controlled rearing experiments involving shift-once and shift-twice transfers of fish of known sex genotype (XX/XY) between feminizing and masculinizing temperatures at different stages of gonadal sex differentiation. Match/mismatch analysis of phenotypic (ovary/testis) and genotypic (absence/presence of the master sex determining gene *amhy*) sex was performed in juveniles to estimate sex reversal rates and the critical period of sex determination for each combination of sex genotype and thermal conditions.

**Results:**

The results show that convergence/divergence between genotypic and environmental signals advances/delays the critical time of sex determination and lowers/raises the degree of environmental sensitiveness, respectively, even when genotypic control is ultimately overridden. This study also provides evidence that ovarian formation is the default state regardless of genotypic sex but commitment to femaleness is a lengthy, passive process requiring absolute seclusion from environmental pro-male stimuli in the span of weeks. Testis formation, in turn, is the alternative state that can be imposed on this default, regardless of genotype, by an extremely short (range of hours) environmental stimulus of sufficient strength at any time before ovarian commitment. We argue that this combination of developmental features increases the likelihood of male development and at the same time may be crucial to avoid ambiguous differentiation under conflicting genotypic/environmental signals in GSD + ESD species.

**Conclusions:**

Overall, the results reveal genotypic sex-dimorphic critical periods of sex determination, show that it is “easier” to make males in pejerrey, and provide clues to understand how GSD + ESD species may prevent discrepant sex determination/differentiation when genotype and environment diverge.

**Supplementary Information:**

The online version contains supplementary material available at 10.1186/s13293-025-00768-7.

## Plain language summary

Genotypic (GSD) and environmental (ESD) sex determination coexist in many species of reptiles, fish, and amphibians. Inherited genotypic signals and environmental factors conceivably interact as pro-testis or pro-ovary signals during sex determination, but how such interactions affect gonadal sex differentiation in these species remains largely unexplained. We used a model gonochoristic fish with coexisting GSD and ESD, the pejerrey *Odontesthes bonariensis*, to examine how synergism and antagonism between sex genotype (XX/XY) and thermal (feminizing/masculinizing) regimes interactively affect environmental sensitiveness and the critical time of environmental sex determination and how genotype-by-environment conflicts are resolved. We show that convergence/divergence between genotypic and environmental signals advances/delays the critical time of sex determination and lowers/raises the degree of environmental sensitiveness, respectively, even when genotypic control is ultimately overridden. The results also evidence that ovarian formation is the default state regardless of genotypic sex and yet that commitment to femaleness is a lengthy, passive process requiring absolute seclusion from environmental pro-male stimuli. Testis formation, in turn, is the alternative state that can be imposed on this default by an extremely short environmental stimulus of sufficient strength at any time before ovarian commitment. We argue that this combination of developmental features is crucial to avoid ambiguous differentiation under conflicting genotypic/environmental signals. Overall, the results reveal genotypic sex-dimorphic critical periods of sex determination, show that it is “easier” to make males in pejerrey, and provide clues to understand how GSD + ESD species prevent discrepant sex determination/differentiation when genotype and environment diverge.

**Supplementary Information:**

The online version contains supplementary material available at 10.1186/s13293-025-00768-7.

## Highlights

- Convergence or divergence between genotypic and environmental signals advances or delays the critical time of sex determination and lowers or raises the degree of environmental sensitiveness, respectively, even when genotypic control is ultimately overridden.
- Ovarian formation is the default state regardless of genotypic sex but commitment to femaleness is a lengthy, passive process requiring absolute seclusion from environmental pro-male stimuli in the span of weeks.
- Testis formation is the alternative state that can be imposed on this default, regardless of genotype, by an extremely short (range of hours) environmental stimulus of sufficient strength at any time before ovarian commitment.
- This combination of features for female and male differentiation increases the likelihood of male development in pejerrey but may also be key to prevent discrepant gonadal differentiation under conflicting genotypic and environmental signals.

**Supplementary Information:**

The online version contains supplementary material available at 10.1186/s13293-025-00768-7.

## Background

Vertebrates exhibit remarkable diversity in morphological architectures, ecological niches, and life history strategies, and yet, species within this evolutionary clade predominantly retain sexual reproduction as a core reproductive strategy. Beyond this seeming unity, however, sex determination in vertebrates utilizes a spectrum of divergent mechanisms spanning from strictly genetically regulated systems (genotypic sex determination, GSD) to environmental cues acting as primary sex determinants (e.g., environmental sex determination, ESD) depending on the clade and species [1–5]. With few exceptions, the GSD mechanism is highly evolutionarily conserved across warm-blooded vertebrates (XX-XY system in mammalians and ZZ-ZW in birds) and under natural conditions external environmental factors are generally unable to substantially alter the genetically programmed sex differentiation trajectory [6, 7]. On the other hand, ectotherm/poikilotherm vertebrate groups (reptiles, amphibia and fish) rely on various forms of GSD (XX-XY, ZZ-ZW, or polygenic systems) and ESD (temperature-, social condition-, photoperiod-dependent sex determination, among other forms) [1, 3, 6]. For long considered as mutually exclusive, there is now ample evidence that these two basic systems coexist to various degrees [1, 8]. A corollary to the coexistence of GSD and ESD in a species is that the gonadal sex of an individual may not match its genetically predisposed sex. Moreover, sex reversal (genotype/phenotype mismatch) in ectothermic vertebrates that combine ESD and GSD usually does not impair viability or sexual development, and such individuals are able to reproduce and thus impact the genic balance and sex ratios of future generations as well as the evolution of sex chromosomes [1, 8–12].

It is generally assumed that sex determination systems act by providing timely signals to the bipotential gonadal primordium that tip the balance towards a pro-testis or pro-ovary direction [13, 14]. Once the initial direction is set, a complex set of endogenous downstream regulatory factors (genes, hormones) further ensures that the path taken is reinforced and, conversely, that the alternative pathway is shut [2, 13, 15]. In species with coexisting GSD and ESD systems, the regulatory interplay between these mechanisms involving epigenetic crosstalk, molecular signaling coordination, and environmental sensory pathway integration, constitutes a pivotal question in evolutionary developmental biology for understanding phenotypic plasticity in sex determination [16]. To begin with, little is known on whether convergence or divergence between genotypic and environmental signals influences the degree of environmental sensitiveness of the primordial gonads during the critical period of sex determination (CPSD). The mounting threat posed by global warming and climate change on the reproduction and sustainability of wild populations of ESD species and the need to understand and counter such effects are also compelling reasons to unravel the mechanistic complexity behind GSD + ESD systems [10, 17–19]. This study uses the pejerrey *Odontesthes bonariensis*, an emerging model organism with both GSD and ESD [20], to investigate the interactive influence through which these two sex determination systems coordinately regulate gonadal sex differentiation.

The pejerrey matches all the biological requirements for performing comparative studies to clarify the temporal interactions between GSD and ESD in a context of environmental relevance. In this species, temperature-dependent sex determination (TSD) predominates as the primary ESD mechanism. In fact, the pejerrey has the most marked TSD known so far among fishes [21], consistently generating all-female and all-male populations when the young experience ecologically relevant low and high temperatures, respectively, during the CPSD [20, 22]. On the other hand, it also possesses a clear Mendelian-inherited sex determination system of the type XX/XY in which the presence of a duplicated copy of the anti-Müllerian hormone gene (*amh*) in the Y chromosome, hence *amhy*, provides sufficient signals to induce testicular formation at intermediate (henceforth “sexually neutral”) temperatures. Interestingly, *amhy* is either redundant or insufficient at the thermal extremes, and in these conditions, it becomes an ideal marker to identify individuals with mismatching phenotypic and genotypic sex [23, 24]. Despite the relatively high “entropy” provided by the coexistence of functional GSD and ESD (TSD), gonadal development in pejerrey is an extremely “clean” process typical of gonochoristic fish. Thus, individuals develop directly as females or males without recognizable intersex stages or pathogenic gonadal features before, during, or after gonadal sex differentiation, suggesting that the processes involved are tightly regulated and “fail-proof” to cope with conflicting endogenous and exogenous signals [20]. The CPSD of pejerrey has been tentatively estimated before the discovery of *amhy* as between 1 and 5 weeks after hatching [22]. In this study, we performed a series of temperature shift experiments to determine with great accuracy the sex reversal rates and the CPSD of the reciprocal genotypes (XX and XY) at masculinizing and feminizing temperatures in order to clarify the existence of GSD + ESD interactions. The results uncover a marked dependency of environmental plasticity of sex determination on the inherited sex genotype and novel features of sex determination in GSD + ESD species that have never been reported before.

## Materials and methods

### Source of larvae and rearing conditions

Pejerrey broodstock of the Yoshida strain were kept under conditions conducive to spawning at the Aquatic Animal Rearing Facilities of TUMSAT, Shinagawa Campus. Fertilized eggs obtained by natural spawning from single pairs of parents of known genotype were used in the experiments. Eggs were incubated at 17℃ and newly hatched larvae were stocked in 60-liter tanks at densities below 15 larvae per liter (approximately 180–250 larvae per tank) to minimize stress and its effect on sex determination following García-Cruz et al. [25]. Tanks were supplied with a small, constant, inflow of brackish water (0.1–0.2% salinity) prepared with dechlorinated tap water and NaCl rock salt to maintain adequate water quality. Illumination with LED fixtures was set at a light intensity of approximately 700 lx at the water surface and a constant photoperiod of 14 h light and 10 h darkness that included dimming periods of one hour at the onset and end of the light phase. Thermal regimes employed in the three experiments are described in detail below. Larvae were fed with *Artemia* nauplii three times per day to satiation until 50 days after hatching and thereafter with a mixture of nauplii and TetraMin flakes (Tetra, Melle, Germany) until termination.

**Fig. 1:**
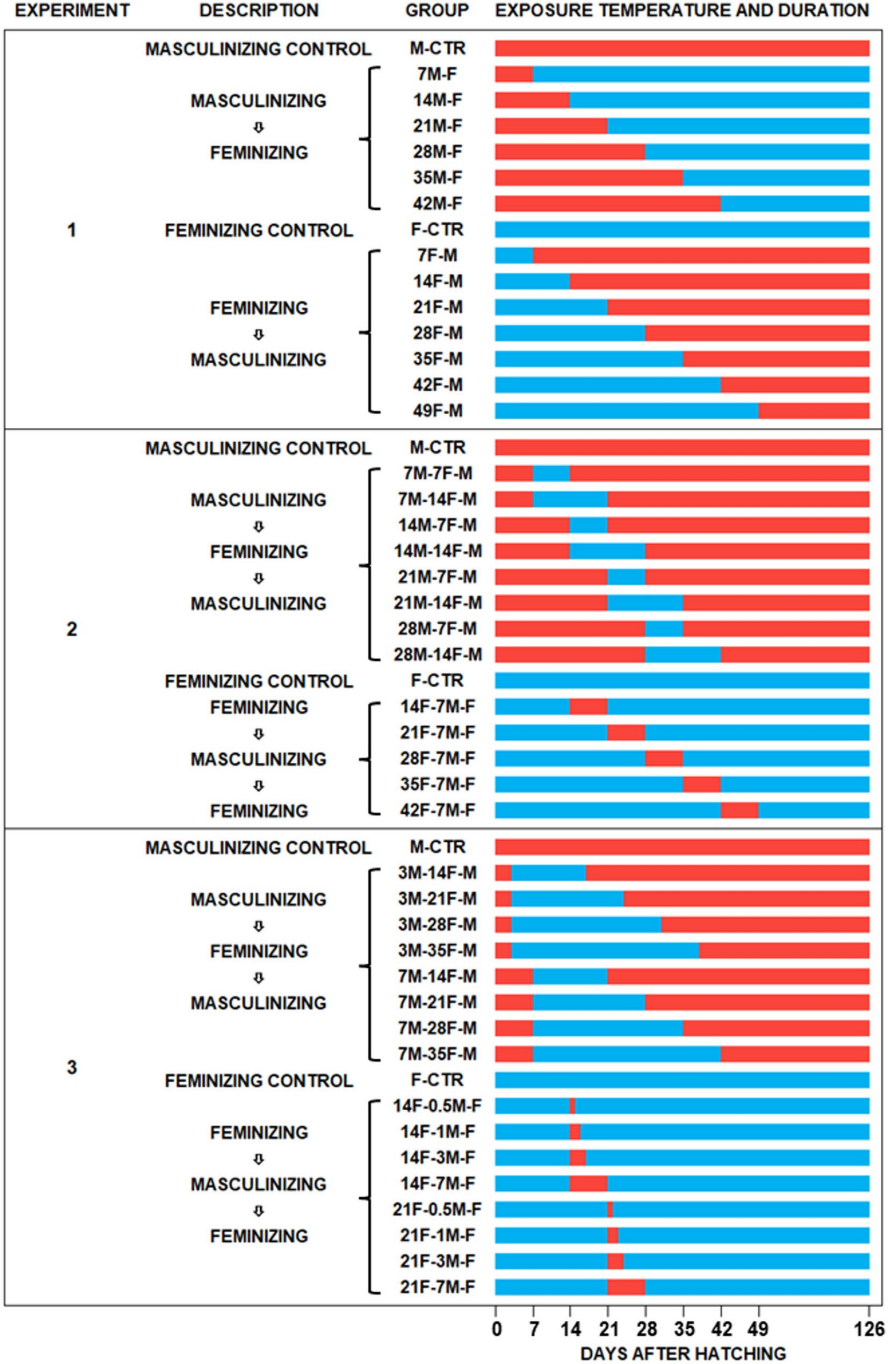
Schedule of the temperature-treatments in experiments 1–3 to define the thermolabile sex determination period of XX and XY pejerrey at feminizing and masculinizing temperatures. Each experiment included dedicated control groups maintained throughout at 17 and 29℃. In experiment 1 (shift-once), six groups were reared first at 29℃ and then transferred permanently to 17℃ at 7, 14, 21, 28, 35, and 42 days after hatching (dah); another seven groups of fish were reared first at the feminizing temperature (17℃, “F”, blue lines) and then transferred permanently to the masculinizing temperature (29℃, “M”, red lines) at 7, 14, 21, 28, 35, 42, and 49 dah. In experiment 2 (shift-twice), eight groups were reared first at 29℃, exposed to 17℃ for 7 or 14 days from 7, 14, 21, and 28 dah, and then returned to 29℃; another five groups were reared first at 17℃, exposed to 29℃ for 7 days from 14, 21, 28, 35, and 42 dah, and then returned to 29℃. In experiment 3 (shift-twice, with refined conditions based on the results of experiment 2), eight groups were reared first at 29℃, exposed to 17℃ for 14, 21, 28, or 35 days from 3 and 7 dah and then returned to 29℃; another eight groups were reared first at 17℃, exposed to 29℃ for 0.5, 1, 3, or 7 days from 14 and 21 dah, and then returned to 17℃

### Temperature shift experiments

The rationale of the temperature shift experiments is that shifting between all-male and all-female producing temperatures at different times would allow inference on whether the sex was determined before or after transfer and therefore on the timing of the CPSD at feminizing and masculinizing conditions [22, 26, 27]. Sex determination in pejerrey is temperature-labile but it is an irreversible process with no secondary sex reversal or formation of intersex gonads even under drastic temperature changes [20]. Three temperature shift experiments were carried out in succession to gradually finetune the CPSD estimation in relation to genotype and temperature (Fig. 1). The timing of the single temperature shifts tested in the first experiment were based on a previous study that broadly defined the CPSD in pejerrey as 1–5 weeks after hatching (wah) at temperatures between 17 and 27 °C [22], at a time when the marker of genotypic sex in pejerrey *(amhy*) was not yet available. For this study, 7 groups of fish were initially reared at 17℃ and then transferred to 29℃ at 7, 14, 21, 28, 35, 42, or 49 days after hatching (dah). Another 6 groups were reared first at 29℃ and then transferred to 17℃ after 7, 14, 21, 28, 35, or 42 days. For the second experiment, a shift-twice experiment, 5 groups were reared first at 17℃ for 14, 21, 28, 38, or 42 days, exposed to 29℃ for 7 days, and then returned to 17℃. Another 8 groups were reared first at 29℃ for 7, 14, 21, or 28 days, exposed to 17℃ for 7 or 14 days, and then returned to 29℃. For the third experiment, 8 groups were reared first at 29℃ for 3 or 7 days, exposed to 17℃ for 14, 21, 28, or 35 days, and then returned to 29℃. Another 8 groups were reared first at 17℃ for 14 or 21 days, exposed to 29℃ for 0.5, 1, 3, or 7 days, and then returned to 17℃. All three experiments included dedicated control groups reared at the feminizing (17℃) and masculinizing (29℃) temperatures from hatching to termination to determine the proportions of temperature-insensitive fish, categorized as GSD fish, in any of the genotypes. Fish were reared for about 4 months until their phenotypic (gonadal) sex could be unambiguously determined by histological analysis and then processed as described below.

### Identification of genotypic and phenotypic sex

Fish sampling in this study followed the protocols outlined in the Guide for the Care and Use of Laboratory Animals from Tokyo University of Marine Science and Technology and the National Research Council of Japan (8th edition). Fish were euthanized as humanely as possible by a combination of hypothermia in ice-water with an anesthetic overdose of 2-Phenoxyethanol (Fujifilm Wako, Osaka, Japan). The caudal fin of each fish was stored in 100% ethanol for use in genomic DNA analysis of the presence of the *amhy* gene as previously described [24]. Individuals with and without the *amhy* gene were classified as XY and XX, respectively. In the first experiment, which inadvertently involved the cross of an XY male with an XY sex-reversed female, the XY and YY genotypes were resolved by qPCR to identify the individuals (YY) with two *amhy* copies [28].

The trunks were used for phenotypic sex determination and were immersed in Bouin’s fixative for 24 h and then stored in 70% ethanol. Materials were subsequently dehydrated through a series of increasing ethanol concentrations, cleared in xylene, and embedded in paraffin (Paraplast Plus, McCormick Scientific, Richmond, IL). Cross sections with a thickness of 6 μm were cut using a microtome (Microm HM 325, Thermo Scientific, Massachusetts) and stained with hematoxylin and eosin. Histological preparations were examined under a microscope (Olympus CH-2, Olympus Corporation, Tokyo, Japan) and categorized as female (ovary) or male (testis) based on histological criteria described elsewhere [29].

### Statistical analysis

The duration of the CPSD for each genotype at a given temperature was inferred from the changes in the percentage of fish with that genotype whose sex determined before or after the temperature shift(s) with progressive delay and/or duration of shifting. Fish were considered as already sex-determined before transfer if their phenotypic sex agreed with the sex induced by the initial temperature; on the contrary, if their phenotypic sex agreed with the destination temperature, they were considered as indetermined at the time of transfer. Sex determination of XY fish in groups temporarily exposed to the feminizing temperature (17 °C) were adjusted by the frequency of XY males in the respective 17 °C controls. This correction was necessary because the Yoshida strain used in this study has a stronger GSD, that makes some of the XY fish to develop as males even at the feminizing temperature [20, 30–32]. Adjustments for feminization of the XY genotype and for masculinization and feminization of the XX genotype were not necessary as their controls were consistently all-male or all-female. The point of 50% sex determination for each combination of genotype and thermal treatments was estimated by logistic regression using GraphPad Prism (v.9; GraphPad Software, San Diego, CA, USA).

## Results

**Fig. 2:**
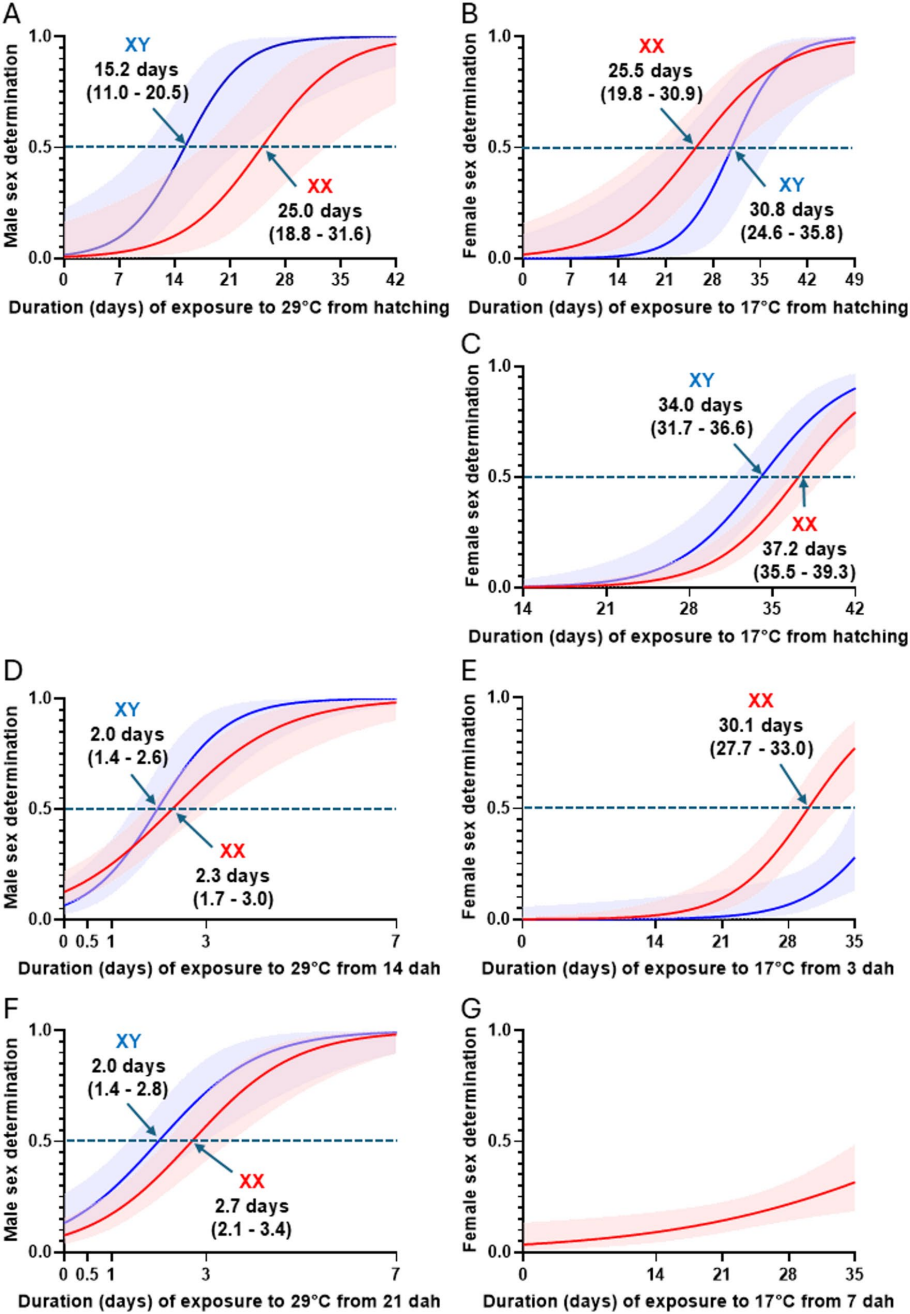
Logistic regressions of the proportions of XX (red) and XY (blue) genotypes undergoing sex determination as females or males in experiments 1–3. The solid lines represent the logistic regression curves and the shaded ribbons the 95% confidence intervals; the time of 50% sex determination for each genotype is indicated when applicable. (**A**, **B**) Proportions of fish that determined as male or female with progressively longer exposure to 29℃ (**A**) or 17℃ (**B**), respectively, from hatching in experiment 1. (**C**): Proportions of fish that determined as females with progressively longer exposure to 17℃ before a one-week exposure to 29℃ in experiment 2 (regression analysis for male determination was not possible as all groups at 29℃ from hatching were 100% masculinized). (**D**, **F**) Proportions of fish that determined as male with 0.5–7-days exposure to 29℃ starting from 14 (**D**) or 21 (**F**) days after hatching (dah) in experiment 3. (**E**, **G**) Proportions of fish that determined as female with 14–35-days exposure to 17℃ starting from 3 (**E**) or 7 (**G**) dah in experiment 3 (regression analysis for XY fish that determined as females by exposure to 17℃ from 7 dah was not possible as there were not enough females)

The evidence gathered in this study includes sex reversal rates and CPSD estimates for fish with the reciprocal sex genotypes (XX/XY) that were exposed transiently to feminizing and masculinizing temperatures at different stages of gonadal development in a series of shift-once and shift-twice rearing experiments. The results of logistic regression of the frequency of sex determination per genotype on the duration of exposure to feminizing and masculinizing temperatures in the first (shift-once) experiment are shown in Figs. 2A and B. Details on the number of individuals classified as phenotypic and genotypic male or female and the ROC curves for the logistic regressions are provided as Supplementary Information (Supplementary Fig. 1 and Table 1). Individuals reared at the masculinizing temperature (29 °C) from hatching that developed testes were first identified by 7 and 21 days for the XY and XX genotypes, respectively. The point of 50% male determination in the XY and XX genotypes at this temperature was reached by 15.2 days (11.0–20.5) and 25.0 days (18.8–31.6), respectively (Fig. 2A). The time of 100% commitment to testis was about 28 and 42 days in XY and XX, respectively. Except for 3 females, most of the YY fish (118/121) were phenotypic male regardless of the rearing temperature, and logistic regressions could not be performed; the 3 YY females were found notably in groups reared for the longest times at the feminizing temperature (17℃) starting either from hatching or shortly thereafter (Supplementary Table 1). Individuals reared at the feminizing temperature (17℃) from hatching that irreversibly determined as phenotypic female appeared first after 14 and 21 dah for the XX and XY genotypes, respectively. The point of 50% female determination in the XX and XY genotypes at this temperature was reached at 25.5 days (95% confidence interval of 19.8–30.9) and 30.8 days (24.6–35.8), respectively (Fig. 2B). Both XX and XY reached 100% female determination at about 35–42 dah.

The results of the second experiment, using a shift-twice approach, are shown in Fig. 2C (see also Supplementary Fig. 1 and Table 2). Experimental groups reared at 29 °C from hatching and exposed to 17 °C for one or two weeks at various timeframes were almost all male regardless of genotype (Supplementary Table 2) and were not amenable to logistic regression analysis. The rates of feminization in groups shifted with progressive delay from the feminizing temperature (17 °C) to a one-week exposure to the masculinizing temperature (29 °C) and then returned to the initial temperature started increasing from 28 dah in both genotypes and reached 50% by 34 and 37.2 days in the XY and XX genotypes, respectively. The proportion of XX and XY individuals developing as females increased with further delays in transfer but never reached 100%.

The results of the third experiment, also using a shift-twice approach but with increasing resolution, are shown in Figs. 2D-G (see also Supplementary Fig. 1 and Table 3). Individuals sex-determined as males were found in all groups shifted temporarily from 17 °C to 29 °C at 14 and 21 dah, including those exposed for only 0.5 days to the masculinizing temperature. The proportion of male determination increased with the duration of exposure to the high temperature and the point of 50% male determination for individuals shifted at 14 dah was reached by 2.0 days (1.4–2.6) and 2.3 days (1.7–3.0) in XY and XX individuals, respectively (Fig. 2D). For those shifted at 21 dah, the point of 50% masculinization was 2.0 days (1.4–2.8) and 2.7 days (2.1–3.4) in XY and XX individuals, respectively (Fig. 2F). For both shifting times, 100% male determination was reached within 7 days at 29 °C regardless of genotype. Groups that were initially reared at 29 °C and temporarily exposed to 17 °C for 14–35 days from 3 to 7 dah were predominantly male, particularly in the later, and never reached 100% female determination (Figs. 2E, G). Individuals feminized by exposure to 17 °C were first identified after 21 and 28 days for the XX and XY genotypes, respectively. The point of 50% feminization of XX fish shifted at 3 dah to 17 °C was reached by 30.1 days (27.7–33.0) (Fig. 2E) but could not be estimated for the XY or for fish transferred at 7 days (Fig. 2G) as these groups were almost all male.

## Discussion

Knowledge on genotypic dimorphism in CPSD of GSD + ESD species, when sex determination is under the influence of endogenous and exogenous influences, is crucial for studies examining the molecular and physiological mechanisms involved. It is also of fundamental importance to model the effects of global environmental issues such as warming and climate change on their reproduction and yet is critically lacking. In our model species the pejerrey, as in other ESD species of fish and reptiles, underlying information on the CPSD pre-dates the discovery of genotypic sex determinants and therefore consists of a gross estimation that lacks sex genotype specificity. Inherited genotypic signals and environmental sex determining factors can conceivably conflict as pro-testis or pro-ovary signals but how this power balance is resolved is largely unknown. Moreover, knowing the exact moment that an individual undergoes irreversible sex commitment will allow precise correlational analyses of genomic, metabolomic, and biochemical parameters with environmental factors to be performed. In this study, we employed the so-called “shift-once” and “shift-twice” thermal protocols [16, 33, 34] to redefine the sensitive window of sex determination in a model GSD + ESD species, the pejerrey, in relation to genotype and temperature. We took advantage that sex determination in this species has a clear male sex genetic determinant, *amhy*, and yet it is strongly canalized under female- and male-inducing temperatures, so that deviation from the expected all-male and all-female sex ratios allows to infer if the sex of an individual was determined before or after transfer, and hence, on the details of the CPSDs for each of the sex genotypes.

**Fig. 3:**
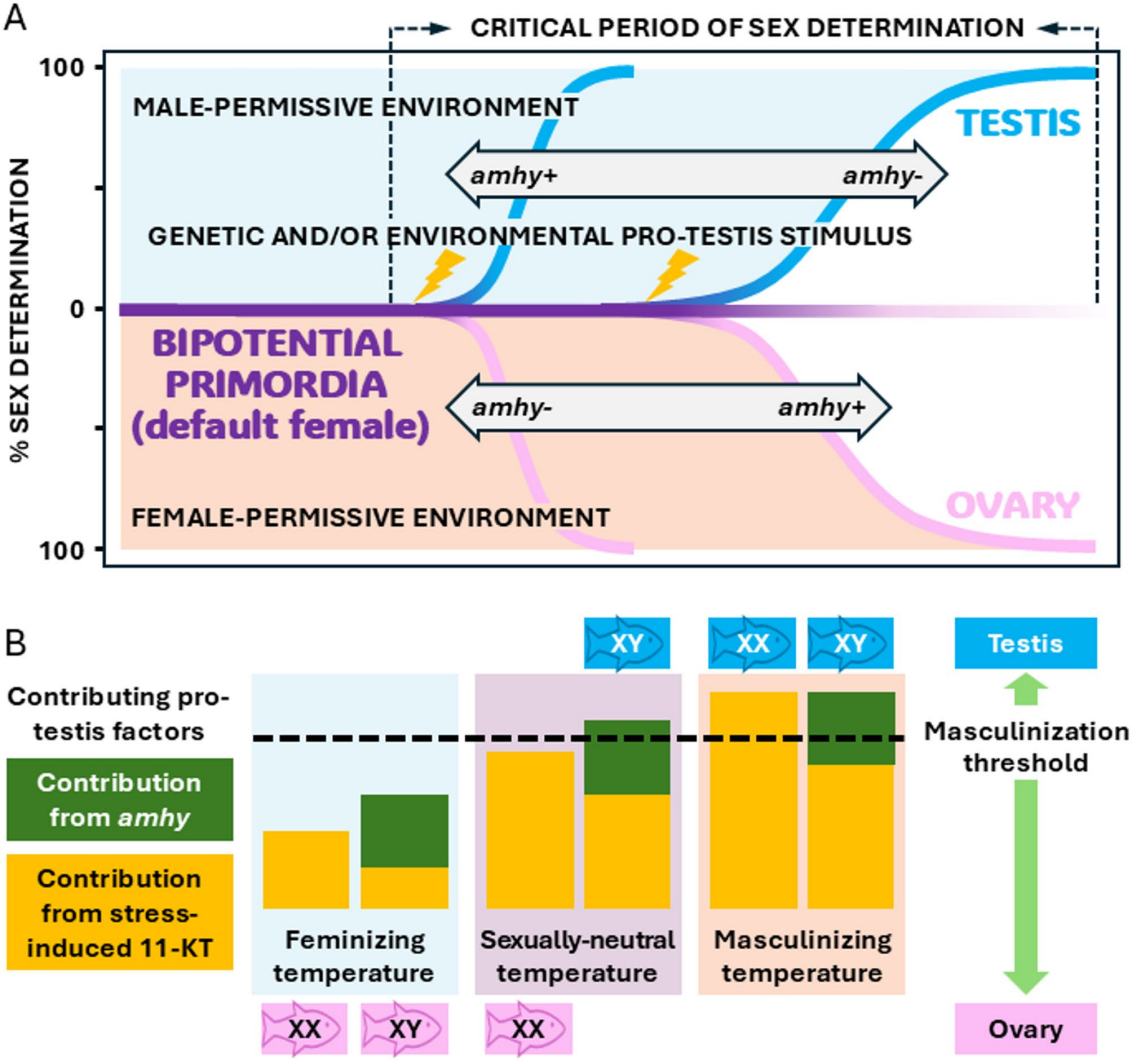
Theoretical representation of the interactive effects of genetic and environmental sex determinants on gonadal fate decision in a GSD+ESD fish species, the pejerrey. (**A**) Testis determination can be imprinted on a bipotential gonadal primordia after reaching a threshold developmental stage (critical period of sex determination) by a timely environmental signal (masculinizing temperature or else) that alone or in combination with the genetic signal (*amhy*+) has sufficient strength to override the default female path before irreversible commitment to ovarian formation, which in turn requires absolute and extended isolation from pro-testis signals to be executed. The timing of commitment to each phenotypic sex is flexible, being advanced or delayed based on the convergence/divergence of genotypic and environmental signals. (**B**) The presence/absence of *amhy* and the level of stress (exemplified here by temperature) during the critical time of sex determination combine to ultimately determine gonadal fate. Note that thermal stress in pejerrey increases with increasing temperature and is genotypically dimorphic, with XX being more sensitive, which transduces in differential androgen (11-KT) levels with temperature and genotype (see references in text)

This study provides fundamental information for understanding the process of sex determination in GSD + ESD species. The first important inference from the results is that endogenous and exogenous signals for sex determination function as vectors that interact to define an individual’s CPSD. Gonadal sex differentiation, as one facet of development in general, is normally advanced with increasing temperature within a physiological range in ectotherms/poikilotherms [35]. A previous shift-once study conducted without the genetic marker of sex (*amhy*) nearly 30 years ago estimated the CPSD of pejerrey as 1–3 weeks and 3–5 weeks after hatching at 27 °C (sex ratios nearly all-male) and 17 °C (all-female), respectively [22]. Like this example with pejerrey, similar studies on other ESD species without a genetic marker of sex also supported the notion that high temperatures accelerate gonadal sex determination but could not dissociate the dynamics and order of sex determination from the underlying influence of temperature on developmental rates [33, 34]. In this context, the inescapable conclusion would be that sex determination is accelerated at higher temperatures in both sexes or that it is faster in the sex favored by high temperature [34]. In contrast, this study shows for the first time that the CPSD can be decoupled from the effects of temperature on developmental rates when genetic and thermal signals diverge, to the extent that the order of sex determination was inverted at low (females earlier by about 5 days) and high (males earlier by about 10 days) temperatures. The decoupling was most evident in the shift-once experiment that involved longer, uninterrupted exposure to low and high temperatures before and after transfers, thus maximizing the difference in developmental rates between the two thermal regimes. Thus, the results clearly indicate the existence of antagonism and synergism between genotypic and environmental factors, delaying or accelerating sex determination when signals diverge or converge, respectively (Fig. 3A). A detailed mechanistic explanation for these findings awaits further investigation. Among the alternatives, the possibility of a differential stress response by the two sex genotypes at different temperatures seems plausible. Sex reversal of the XX genotype in pejerrey is known to involve a temperature-dependent stress reaction leading to cortisol release and the collateral synthesis of the potent masculinizing steroid hormone 11-ketotestosterone during cortisol deactivation by the enzyme 11β-HSD [20, 36, 37]. A tempting conjecture is therefore that the combined absence of the genetic male driver *amhy* and low stress/cortisol/11-ketotestosterone levels at the low temperature comparatively potentiate ovarian development in XX fish whereas their combined presence has the reciprocal effect on testicular development in XY at the high temperature (Fig. 3B).

A second important line of findings is related to the dynamics of loss of sexual bipotentiality of the gonads and the contrasting requirements for sex determination of females and males. Female determination under a stable feminizing regime from hatching was centered at 3–4 weeks but in some fish it took as long as 6–7 weeks. Moreover, formation of ovaries seemed to be conditional to the absence of an environmental pro-male signal (masculinizing temperature) for a minimum of 2–4 consecutive weeks, as can be seen most clearly in groups exposed transiently, but not sufficiently to induce masculinization, to 29 °C before or immediately after exposure to the feminizing temperature. The wide range in the timing of female determination reflects in part the interactions between genotype and temperature discussed above, with shorter and longer periods for XX and XY, respectively, but may also reflect individual differences in growth rates. In the pejerrey, as in other fish species, development is more closely related to body size than age [38]. We therefore assume that fast growing fish achieved a hitherto undetermined developmental threshold for sex determination earlier than slow growing fish, and that individual differences are conceivably magnified in the lowest temperatures. In contrast to the slow definition of ovaries, induction of testis differentiation by high temperature proceeded very fast. Fish reared at the masculinizing temperature from hatching were irreversibly sex-determined as males within 1–3 weeks, again with genotype- and probably growth rate-dependence. The most intriguing finding of this study, however, was that testis could be induced by transient exposure of larvae from a feminizing background to a masculinizing temperature as short as 0.5 days. The response to the short masculinizing trigger was essentially similar for exposures beginning at 14 or 21 dah in the feminizing temperature, suggesting the lack of any significant loss of environmental sensitiveness of the undetermined gonads, and therefore, that the gonads have not substantially progressed towards a female outcome that could offer resistance to masculinization during this period. However, we cannot rule that incipient stages of ovarian formation, such as with longer exposures to the feminizing temperature, might still retain the ability to be masculinized. If such gonads resist masculinization to some degree, it could explain part of the individual variation in female CPSDs discussed above, more so if the resistance is genotypic sex-dimorphic. Ongoing analysis of the expression profiles of sex-related genes at this critical period should help clarify the existence of a molecular threshold between bipotentiality and sex commitment in pejerrey. Likewise, shorter exposures to high temperatures or alternative forms of stress are being tested to clarify the minimum effective trigger for masculinization. Parallel studies are also scrutinizing the relative importance of size and age as developmental parameters affecting sex determination in our model in relation to genotype and temperature.

The finding that commitment to testis determination in pejerrey is an extremely short event has parallels in sex determination of other vertebrates. The expression of *sry*, the genetic trigger of male sex determinant in most mammals that is harbored on the Y chromosome, occurs only in the span of hours and yet it suffices to irreversibly set the course for testicular development through concerted action of downstream regulatory factors that shut off completely the female pathway [2, 13, 14, 39]. Information on ESD species is limited, but at least in two species of fish [40, 41] and two species of turtles [42, 43], it has been suggested that sex determination may be a short event too. Interestingly, high temperature favors the formation of males in the former, as it is in pejerrey, while in the latter it is the opposite. There is also a report of a high proportion of males in coho salmon when embryos experienced high temperature during a very short period in early development [44]. Taken together with our results, these examples suggest that sex determination may be an active, short event in one of the sexes and lengthy in the other in more species. This concept hinges on the existence of a default sex with a nearly autonomous development and the reciprocal, where a trigger, internal (genetic), external (environmental), or both is necessary. In mammals (male heterogamety) and birds (female heterogamety), females and males are thought to be the default sex, respectively, during early development [13, 14, 45]. In pejerrey, which has the XX/XY system, it has been shown that ovarian formation is the default state based on the results of morphological and molecular studies [20, 46]. Examination of other species with known patterns of GSD, alone or in combination with ESD, might shed light on the existence of a relationship between the chromosomal system of sex determination and the putative default sex.

In this study, most gonads lost sexual bipotentiality after 1 and 4 weeks from hatching at 29 and 17 °C, respectively. This is much earlier than the first signs of morphological differentiation, that is, 3 weeks for testes at 29 °C and 7 weeks for ovaries at 17 °C [29]. In pejerrey, *amhy* is expressed early and transiently in XY fish at both masculinizing and feminizing temperatures but the expression window is much shorter in the former (ca. 1 week) than in the latter (ca. 3 weeks) [30]. Thus, there is a close temporal coincidence between the end of *amhy* expression and the thermolabile period, suggesting a converging mechanism between genotypic and environmental sex determination as already noted in other species [47]. In addition, that a single, short switch suffices to irreversibly induce masculinization suggests a tightly controlled mechanism. Keeping one sex as the default and limiting active sex determination to the alternate sex as a “one switch”, making it short and providing accessory mechanisms to instantaneously and irreversibly shut off the alternative pathway once the switch is activated seems to be a rather logical, fail-proof approach to fend off discrepant differentiation within the gonads in case of conflicting genetic and environmental signals during sex determination of GSD + ESD species such as the pejerrey. However, as seen in mammals, where the initial steps of sex determination are highly canalized, these features are also shared characteristic even with homeotherms. The evolutionary order of GSD and ESD is still debatable [8] and in this context our findings could be invoked as evidence favoring ESD as the ancestral form. Our results could be relevant also to the observed association between slow development/poor growth rates, malnutrition, and starvation and the formation of males in environmentally sex-determined fish [48–51]. We conjecture that simple probability theory in the context of the current findings might explain a large fraction of the female-to-male sex reversals. For example, if a short stimulus given after the gonads have passed a developmental threshold for testis determination but have not developed enough for full ovarian commitment suffices to induce masculinization, any factor delaying development, including malnourishment, should proportionally increase the probability of male sex reversal. Thus, poor growth may lead to lengthening of the CPSD, increasing the probability that an individual may receive the masculinizing stimulus while it is still sensitive.

As a corollary to the short signaling required for masculinization, the higher stress-sensitiveness of XX fish during the CPSD than XY [25, 52], and the natural masculinization drive provided by *amhy* in XY fish, it can be concluded that by virtue of genomic-, environmental-, or their combined stimuli, in pejerrey it is much easier to become a male than a female. This may be the reason why field studies in pejerrey and in a closely related species with similar characteristics, the cobaltcap silverside, show that sex reversal of XY to females is a much rarer event compared to sex reversal of XX to males [53]. In a GSD + ESD reptile with male heterogamety, the Alpine skink *Bassiana duperreyi*, only XX sex-reversed males were found in the wild [54]. Interestingly, the reciprocal was observed in a field study of the bearded dragon *Pogona vitticeps*, a species combining female heterogamety and high temperature-induced female sex reversal with a sharp pivotal threshold, whereby Holleley et al. [10] found only ZZ sex-reversed females in addition to normal ZZ males and ZW females.

## Conclusions

Using the pejerrey as a model species that combines both genotypic and environmental sex determination, this study reveals that the presence of an inherited sex determinant (*amhy*) can impact greatly the degree and timing of environmental sensitiveness of sex determination in GSD + ESD species, even when it is ultimately overridden by the environment. The interactive effects of genotype and environment in pejerrey were so marked that not only the critical time of sex determination could be advanced or delayed by several days, but its order was inverted between feminizing (females earlier) and masculinizing (males earlier) temperatures depending on the divergence or convergence of pro-testis and pro-ovary genotypic and environmental signals. Likewise, it is clear from the results that the presence or absence of the master sex determining gene affects the thermal threshold for masculinization (see graphical representation in Fig. 3B). Equally important, this study reveals for the first time the contrasting requirements and dynamics for ovarian and testicular commitment. Thus, while ovarian formation is the default state regardless of genotypic sex but requires several weeks under strictly female-permissive conditions to become established, induction of testis formation requires only a short pulse of masculinizing signals currently estimated to be effective in the range of hours.

### Perspectives and significance

Careful consideration of the different dynamics for ovarian and testicular commitment revealed in this study, weeks vs. hours, respectively, and how they are affected by genotype/environment interactions may be key for studies aiming to clarify the molecular and endocrine mechanisms of genotypic and environmental sex determination and particularly crucial for correlative analysis of environmental factors and sex ratios of wild populations. The realization that a short switch from potentially multiple sources suffices to induce masculinization in combination with the genotypic differences discussed above also underscores the complexity of establishing a correlation between a particular environmental factor such as temperature and sex determination (sex reversal/sex ratios) in wild populations. Indeed, this may constitute a formidable challenge when modeling the effects of climate change on ESD species that are already freely moving during their critical period of sex determination such as fish. On the other hand, the novel concepts tentatively derived from this study in pejerrey, that is of the putative homogametic sex being the default outcome during sex determination, that sex determination in the homogametic sex is a lengthy process requiring stable conditions and isolation from stimuli, that the putative heterogametic sex is induced by a short switch of alternative sources (genetic and environmental), and therefore that sex reversals from the default status are more common, may apply also to other GSD + ESD biological systems. Careful testing of these hypotheses could provide a catalyst for the exploration of the underlying mechanisms of sex determination in vertebrates.

## Supplementary Information

Table S1: Number of individuals classified as genotypic and phenotypic male or female in the various treatments of experiment 1. Table S2: Number of individuals classified as genotypic and phenotypic male or female in the various treatments of experiment 2. Table S3: Number of individuals classified as genotypic and phenotypic male or female in the various treatments of experiment 3.

Fig. S1: Receiver-operating characteristic (ROC) curves for the logistic regressions shown in Fig. 2.

## Data Availability

Additional information on the experimental protocols is available from the corresponding authors on reasonable request. No datasets were generated or analyzed during the current study.

## References

[1] Perrin N. Sex reversal: a fountain of youth for sex chromosomes? Evolution. 2009;63:3043–49. 19744117 10.1111/j.1558-5646.2009.00837.x

[2] Angelopoulou R, Lavranos G, Manolakou P. Sex determination strategies in 2012: towards a common regulatory model? Reprod Biol Endocrinol. 2012;10:13. 22357269 10.1186/1477-7827-10-13 PMC3311596

[3] Gamble T, Zarkower D. Sex determination. Curr Biol. 2012;22:257–62. 10.1016/j.cub.2012.02.054 PMC11003734 22537624

[4] Kikuchi K, Hamaguchi S. Novel sex-determining genes in fish and sex chromosome evolution. Dev Dyn. 2013;242:339–53. 23335327 10.1002/dvdy.23927

[5] Capel B. Vertebrate sex determination: evolutionary plasticity of a fundamental switch. Nat Rev Genet. 2017;18:675–89. 28804140 10.1038/nrg.2017.60

[6] Marshall Graves JA. Weird animal genomes and the evolution of vertebrate sex and sex chromosomes. Annu Rev Genet. 2008;42:565–86. 18983263 10.1146/annurev.genet.42.110807.091714

[7] Saunders PA, Veyrunes F. Unusual mammalian sex determination systems: a cabinet of curiosities. Genes. 2021;12:1770. 34828376 10.3390/genes12111770 PMC8617835

[8] Bull JJ. Sex determination: are two mechanisms better than one? J Biosci. 2008;32:5–8. 10.1007/s12038-008-0016-9 18376065

[9] Donelson J, Munday PL. Transgenerational plasticity mitigates the impact of global warming to offspring sex ratios. Glob Chang Biol. 2015;21:2954–62. 25820432 10.1111/gcb.12912

[10] Holleley CE, O’Meally D, Sarre SD, Marshall Graves JA, Ezaz T, Matsubara K, et al. Sex reversal triggers the rapid transition from genetic to temperature-dependent sex. Nature. 2015;523:79–82. 26135451 10.1038/nature14574

[11] Wedekind C. Demographic and genetic consequences of disturbed sex determination. Philos Trans R Soc Lond B Biol Sci. 2017;372:20160326. 28760767 10.1098/rstb.2016.0326 PMC5540866

[12] Santidrián Tomillo P. Re-equilibrating sex ratios: adjustment of reaction norms in species with temperature-dependent sex determination. Glob Chang Biol. 2024;30:e17568. 39492691 10.1111/gcb.17568

[13] Camerino G, Parma P, Radi O, Valentini S. Sex determination and sex reversal. Curr Op Genet Dev. 2006;16:289–92. 16647843 10.1016/j.gde.2006.04.014

[14] Nicol B, Estermann MA, Yao HH-C, Mellouk N. Becoming female: ovarian differentiation from an evolutionary perspective. Front Cell Dev Biol. 2022;10:944776. 36158204 10.3389/fcell.2022.944776 PMC9490121

[15] Lundgaard Riis M, Jørgensen A. Deciphering sex-specific differentiation of human fetal gonads: insight from experimental models. Front Cell Dev Biol. 2022;10:902082. 35721511 10.3389/fcell.2022.902082 PMC9201387

[16] Valenzuela N. Introduction and Conclusions. In: Valenzuela N, Lance VA, editors. Temperature-dependent sex determination in vertebrates. Washington DC: Smithsonian Institution; 2004. pp. 157–160.

[17] Walther GR, Post E, Convey P, Menzel A, Parmesan C, Beebee TJC, Fromentin J-M, Hoegh-Guldberg O, Bairlein F. Ecological responses to recent climate change. Nature. 2002;416:389–95. 11919621 10.1038/416389a

[18] Hulin V, Delmas V, Girondot M, Godfrey MH, Guillon JM. Temperature-dependent sex determination and global change: are some species at greater risk? Oecologia. 2009;160:493–506. 19277719 10.1007/s00442-009-1313-1

[19] Valenzuela N, Literman R, Neuwald JL, Mizoguchi B, Iverson JB, Riley JL, et al. Extreme thermal fluctuations from climate change unexpectedly accelerate demographic collapse of vertebrates with temperature-dependent sex determination. Sci Rep. 2019;9:4254. 30862793 10.1038/s41598-019-40597-4 PMC6414666

[20] Strüssmann CA, Yamamoto Y, Hattori RS, Fernandino JI, Somoza GM. Where the ends meet: an overview of sex determination in atheriniform fishes. Sex Dev. 2021;15:80–92. 33951664 10.1159/000515191

[21] Ospina-Álvarez N, Piferrer F. Temperature-dependent sex determination in fish revisited: prevalence, a single sex ratio response pattern, and possible effects of climate change. PLoS ONE. 2008;3:e2837. 18665231 10.1371/journal.pone.0002837 PMC2481392

[22] Strüssmann CA, Saito T, Usui M, Yamada H, Takashima F. Thermal thresholds and critical period of thermolabile sex determination in two atherinid fishes, *Odontesthes bonariensis* and *Patagonina hatcheri*. J Exp Zool. 1997;278:167–77.

[23] Hattori RS, Murai Y, Oura M, Masuda S, Majhi SK, Sakamoto T, et al. A Y-linked anti-Müllerian hormone duplication takes over a critical role in sex determination. Proc Natl Acad Sci USA. 2012;109:2955–59. 10.1073/pnas.1018392109 PMC3286941 22323585

[24] Yamamoto Y, Zhang Y, Sarida M, Hattori RS, Strüssmann CA. Coexistence of genotypic and temperature-dependent sex determination in Pejerrey *Odontesthes bonariensis*. PLoS ONE. 2014;9:e102574. 25036903 10.1371/journal.pone.0102574 PMC4103838

[25] García-Cruz EL, Yamamoto Y, Hattori RS, de Vasconcelos LM, Yokota M, Strüssmann CA. Crowding stress during the period of sex determination causes masculinization in Pejerrey *Odontesthes bonariensis*, a fish with temperature-dependent sex determination. Comp Biochem Physiol Mol Integr Physiol. 2020;245:110701. 10.1016/j.cbpa.2020.110701 32298809

[26] Conover DO, Fleisher MH. Temperature-sensitive period of sex determination in the Atlantic silverside, *Menidia Menidia*. Can J Fish Aquat Sci. 1986;43:514–20.

[27] Conover DO, Heins SW. The environmental and genetic components of sex ratio in *Menidia Menidia* (Pisces: Atherinidae). Copeia. 1987;1987:732–43.

[28] Hattori RS, Tashiro S, Zhang Y, Kakuta N, Yokota M, Strüssmann CA, et al. Demonstration of viability and fertility and development of a molecular tool to identify YY supermales in a fish with both genotypic and environmental sex determination. Ecol Evol. 2018;8:7522–28. 30151167 10.1002/ece3.4148 PMC6106197

[29] Ito LS, Yamashita M, Takashima F, Strüssmann CA. Dynamics and histological characteristics of gonadal sex differentiation in Pejerrey (*Odontesthes bonariensis*) at feminizing and masculinizing temperatures. J Exp Zool Comp Exp Biol. 2005;303:504–14. 10.1002/jez.a.159 15880765

[30] Zhang Y, Hattori RS, Sarida M, García Cruz EL, Strüssmann CA, Yamamoto Y. Expression profiles of *Amhy* and major sex-related genes during gonadal sex differentiation and their relation with genotypic and temperature-dependent sex determination in Pejerrey *Odontesthes bonariensis*. Gen Comp Endocrinol. 2018;265:196–201. 29550552 10.1016/j.ygcen.2018.03.013

[31] Strüssmann CA, Calsina Cota JC, Phonlor G, Higuchi H, Takashima F. Temperature effects on sex differentiation of two South American atherinids, *Odontesthes Argentinensis* and *Patagonina hatcheri*. Environ Biol Fishes. 1996;47:143–54.

[32] Strüssmann CA, Moriyama S, Hanke EF, Calsina Cota JC, Takashima F. Evidence of thermolabile sex determination in Pejerrey. J Fish Biol. 1996;48:643–51.

[33] Valenzuela N. Constant, shift, and natural temperature effects on sex determination in *Podocnemis expansa* turtles. Ecology. 2001;82:3010–24.

[34] Rhen T, Fagerlie R, Schroeder A, CrossleyII DA, Lang JW. Molecular and morphological differentiation of testes and ovaries in relation to the thermosensitive period of gonad development in the snapping turtle, *Chelydra serpentina*. Differentiation. 2015;89:31–41. 25662229 10.1016/j.diff.2014.12.007

[35] McCue MD. General effects of temperature on animal biology. In: Valenzuela N, Lance VA, editors. Temperature-dependent sex determination in vertebrates. Washington DC: Smithsonian Institution; 2004. pp. 71–8.

[36] Hattori RS, Fernandino JI, Kishii A, Kimura H, Kinno K, Oura M, et al. Cortisol-induced masculinization: does thermal stress affect gonadal fate in pejerrey, a teleost fish with temperature-dependent sex determination? PLoS ONE. 2009;4:e6548. 19662094 10.1371/journal.pone.0006548 PMC2717333

[37] Fernandino JI, Hattori RS, Kishii A, Strüssmann CA, Somoza GM. The cortisol and androgen pathways cross talk in high temperature-induced masculinization: the 11-hydroxysteroid dehydrogenase as a key enzyme. Endocrinology. 2012;153:6003–11. 23041673 10.1210/en.2012-1517

[38] Chalde T, Fernández DA, Cussac VE, Somoza GM. The effect of rearing temperature in larval development of pejerrey, *Odontesthes bonariensis* – morphological indicators of development. Neotrop Ichthyol. 2011;9:745–56.

[39] Hiramatsu R, Matoba S, Kanai-Azuma M, Tsunekawa N, Katoh-Fukui Y, Kurohmaru M, Morohashi KI, Wilhelm D, Koopman P, Kanai Y. A critical time window of *Sry* action in gonadal sex determination in mice. Development. 2009;136:129–38. 19036799 10.1242/dev.029587

[40] Santi S, Gennotte V, Toguyeni A, Mélard C, Antoine N, Rougeot C. Thermosensitivity of the sex differentiation process in the African catfish, *Clarias gariepinus*: determination of the thermosensitive period. Aquaculture. 2016;455:73–80.

[41] Du WX, Li Z, Wang T, Ding M, Wang MT, Miao C, Zhang XJ, Wang Y, Wang ZW, Zhou L, Gui JF, Li XY. Gonadal temperature-sensitive gene identification in gynogenetic Gibel carp (*Carassius Gibelio*) with temperature-dependent sex determination. Aquaculture. 2026;611:743065.

[42] Valenzuela N, Botero R, Martínez E. Field study of sex determination in *Podocnemis expansa* from Colombian Amazonia. Herpetologica. 1997;53:390–8.

[43] Carter AW, Paitz RT, Bowden RM. The devil is in the details: identifying aspects of temperature variation that underlie sex determination in species with TSD. Integr Comp Biol. 2019;59:1081–8. 31095337 10.1093/icb/icz036 PMC6797911

[44] Craig JK, Foote CJ, Wood CC. Evidence for temperature-dependent sex determination in Sockeye salmon (*Oncorhynchus nerka*). Can J Fish Aquat Sci. 1996;53:141–7.

[45] Place AR, Lance VA. The temperature-dependent sex determination drama: same cast, different stars. In: Valenzuela N, Lance VA, editors. Temperature-dependent sex determination in vertebrates. Washington DC: Smithsonian Institution; 2004. pp. 99–110.

[46] Strüssmann CA, Ito LS. Where does gonadal sex differentiation begin? Gradient of histological sex differentiation in the gonads of pejerrey, *Odontesthes bonariensis* (Pisces, Atherinidae). J Morphol. 2005;265:190–6. 15971266 10.1002/jmor.10351

[47] Whiteley SL, Holleley CE, Wagner S, Blackburn F, Deveson IW, Marshall Graves JA, et al. Two transcriptionally distinct pathways drive female development in a reptile with both genetic and temperature dependent sex determination. PLoS Genet. 2021;17:e1009465. 33857129 10.1371/journal.pgen.1009465 PMC8049264

[48] Warner DA, Lovern MB, Shine R. Maternal nutrition affects reproductive output and sex allocation in a lizard with environmental sex determination. Proc R Soc Lond B Biol Sci. 2007;274:883–90. 10.1098/rspb.2006.0105 PMC2093968 17251109

[49] Sakae Y, Oikawa A, Sugiura Y, Mita M, Nakamura S, Nishimura T, et al. Starvation causes female-to-male sex reversal through lipid metabolism in the teleost fish, Medaka (*Oryzias latipes*). Biol Open. 2020;9:bio050054. 32265199 10.1242/bio.050054 PMC7132775

[50] Sakae Y, Tanaka M. Metabolism and sex differentiation in animals from a starvation perspective. Sex Dev. 2021;15:168–78. 34284403 10.1159/000515281

[51] Geffroy B. Energy as the cornerstone of environmentally driven sex allocation. Trends Endocrinol Metab. 2022;33:670–9. 35934660 10.1016/j.tem.2022.07.002

[52] Torres-Martínez A, Hattori RS, Fernandino JF, Somoza GM, Song DH, Masuda Y, et al. Temperature- and genotype-dependent stress response and activation of the hypothalamus-pituitary-interrenal axis during temperature-induced sex reversal in Pejerrey *Odontesthes bonariensis*, a species with genotypic and environmental sex determination. Mol Cell Endocrinol. 2024;582:112114. 38008372 10.1016/j.mce.2023.112114

[53] Miyoshi K, Hattori RS, Strüssmann CA, Yokota M, Yamamoto Y. Phenotypic/genotypic sex mismatches and temperature-dependent sex determination in a wild population of an old world atherinid, the Cobaltcap silverside *Hypoatherina Tsurugae*. Mol Ecol. 2020;29:2349–58. 32474976 10.1111/mec.15490

[54] Dissanayake DSB, Holleley CE, Deakin JE, Georges A. High elevation increases the risk of Y chromosome loss in alpine Skink populations with sex reversal. Heredity. 2021;126:805–16. 33526811 10.1038/s41437-021-00406-z PMC8102603

