# Supplementary material for: Genotype-by-environment interactive effects and conflict solving during gonadal sex differentiation of pejerrey Odontesthes bonariensis, a fish with dual genotypic/environmental sex determination
Source: Biol Sex Differ. 2025 Oct 16;16:79. doi: 10.1186/s13293-025-00768-7 (PMC12532474; doi:10.1186/s13293-025-00768-7)
Supplement: Supplementary file 1 — Table S1: Number of individuals classified as genotypic and phenotypic male or female in the various treatments of experiment 1. Table S2: Number of individuals classified as genotypic and phenotypic male or female in the various treatments of experiment 2. Table S3: Number of individuals classified as genotypic and phenotypic male or female in the various treatments of experiment 3. [file 13293_2025_768_MOESM1_ESM.docx]

**Supplementary Information**

**Supplementary Table S1.** Number of individuals classified as genotypic and phenotypic male or female in the various treatments of experiment 1.

| Description | Group^*1^ | Phenotypic sex | | | | | |
| --- | --- | --- | --- | --- | --- | --- | --- |
|  |  | Female | | | Male | | |
|  |  | Genotypic sex^*2^ | | | | | |
|  |  | XX | XY | YY | XX | XY | YY |
| Masculinizing control | M-CTR | 0 | 0 | 0 | 7 | 16 | 8 |
| Masculinizing  ↓  Feminizing | 7M-F | 8 | 8 | 1 | 0 | 11 | 4 |
|  | 14M-F | 4 | 2 | 0 | 0 | 15 | 6 |
|  | 21M-F | 1 | 0 | 0 | 1 | 16 | 9 |
|  | 28M-F | 1 | 1 | 0 | 4 | 6 | 5 |
|  | 35M-F | 0 | 0 | 0 | 3 | 10 | 8 |
|  | 42M-F | 1 | 0 | 0 | 4 | 18 | 9 |
| Feminizing control | F-CTR | 7 | 6 | 1 | 0 | 15 | 17 |
| Feminizing  ↓  Masculinizing | 7F-M | 0 | 0 | 0 | 3 | 14 | 8 |
|  | 14F-M | 2 | 0 | 0 | 5 | 12 | 6 |
|  | 21F-M | 3 | 1 | 0 | 2 | 8 | 4 |
|  | 28F-M | 1 | 0 | 0 | 7 | 10 | 15 |
|  | 35F-M | 6 | 2 | 0 | 0 | 8 | 9 |
|  | 42F-M | 7 | 9 | 0 | 0 | 5 | 4 |
|  | 49F-M | 5 | 10 | 1 | 0 | 7 | 6 |

^*1^ Group names refer to experiment 1 in Figure 1; briefly, the numbers represent the period (days) spent at the feminizing (F) or masculinizing (M) temperature from hatching until transfer to the alternate condition. Controls were reared throughout at masculinizing or feminizing conditions.

^*2^ This experiment was conducted with progeny from a cross of a XY male and a sex-reversed XY female, hence the presence of the YY genotype.

**Supplementary Table S2.** Number of individuals classified as genotypic and phenotypic male or female in the various treatments of experiment 2.

| Description | Group^*1^ | Phenotypic sex | | | |
| --- | --- | --- | --- | --- | --- |
|  |  | Female | | Male | |
|  |  | Genotypic sex | | | |
|  |  | XX | XY | XX | XY |
| Masculinizing control | M-CTR | 0 | 0 | 22 | 26 |
| Masculinizing  ↓  Feminizing  ↓  Masculinizing | 7M-7F-M | 0 | 0 | 22 | 28 |
|  | 7M-14F-M | 0 | 0 | 24 | 40 |
|  | 14M-7F-M | 0 | 0 | 27 | 21 |
|  | 14M-14F-M | 1 | 0 | 22 | 24 |
|  | 21M-7F-M | 0 | 0 | 23 | 24 |
|  | 21M-14F-M | 0 | 0 | 24 | 30 |
|  | 28M-7F-M | 0 | 0 | 20 | 20 |
|  | 28M-14F-M | 0 | 0 | 28 | 32 |
| Feminizing control | F-CTR | 26 | 21 | 0 | 25 |
| Feminizing  ↓  Masculinizing  ↓  Feminizing | 14F-7M-F | 0 | 1 | 25 | 21 |
|  | 21F-7M-F | 0 | 0 | 32 | 40 |
|  | 28F-7M-F | 1 | 1 | 50 | 44 |
|  | 35F-7M-F | 19 | 13 | 17 | 29 |
|  | 42F-7M-F | 18 | 14 | 9 | 22 |

^*1^ Group names refer to experiment 2 in Figure 1; briefly, the numbers represent the period (days) spent at the feminizing (F) or masculinizing (M) temperature from hatching until the first transfer to the alternate condition and between the first and second transfers. Controls were reared throughout at masculinizing or feminizing conditions.

**Supplementary Table S3.** Number of individuals classified as genotypic and phenotypic male or female in the various treatments of experiment 3.

| Description | Group^*1^ | Phenotypic sex | | | |
| --- | --- | --- | --- | --- | --- |
|  |  | Female | | Male | |
|  |  | Genotypic sex | | | |
|  |  | XX | XY | XX | XY |
| Masculinizing control | M-CTR | 0 | 0 | 28 | 23 |
| Masculinizing  ↓  Feminizing  ↓  Masculinizing | 3M-14F-M | 0 | 0 | 26 | 24 |
|  | 3M-21F-M | 2 | 0 | 21 | 27 |
|  | 3M-28F-M | 9 | 2 | 10 | 29 |
|  | 3M-35F-M | 15 | 5 | 6 | 24 |
|  | 7M-14F-M | 2 | 0 | 18 | 30 |
|  | 7M-21F-M | 4 | 0 | 22 | 24 |
|  | 7M-28F-M | 9 | 0 | 15 | 26 |
|  | 7M-35F-M | 4 | 2 | 19 | 25 |
| Feminizing control | F-CTR | 22 | 18 | 1 | 9 |
| Feminizing  ↓  Masculinizing  ↓  Feminizing | 14F-0.5M-F | 21 | 14 | 6 | 9 |
|  | 14F-1M-F | 18 | 9 | 9 | 14 |
|  | 14F-3M-F | 11 | 4 | 15 | 20 |
|  | 14F-7M-F | 0 | 0 | 24 | 26 |
|  | 21F-0.5M-F | 25 | 8 | 4 | 13 |
|  | 21F-1M-F | 22 | 11 | 6 | 11 |
|  | 21F-3M-F | 13 | 5 | 14 | 18 |
|  | 21F-7M-F | 0 | 0 | 22 | 28 |

^*1^ Group names refer to experiment 3 in Figure 1; briefly, the numbers represent the period (days) spent at the feminizing (F) or masculinizing (M) temperature from hatching until the first transfer to the alternate condition and between the first and second transfers. Controls were reared throughout at masculinizing or feminizing conditions.

**Supplementary Figure S1.** Receiver-operating characteristic (ROC) curves for the logistic regressions shown in Figure 2. Only the least significant value of P in comparison to random classification (see explanation below) among the two genotypes for each panel is shown. The X and Y axes represent the specificity (false positive rate, the proportion of false positives among actual negatives), and the sensitivity (true positive rate, the proportion of true positives among actual positives) for each model. The diagonal dashed line (Y = X) represents a random classifier as a baseline for comparison. A curve above this line indicates better-than-random classification performance (GraphPad Prism, v. 9.5.1).
