# Supplementary figures and images for: Genotype-by-environment interactive effects and conflict solving during gonadal sex differentiation of pejerrey Odontesthes bonariensis, a fish with dual genotypic/environmental sex determination
Source: Biol Sex Differ. 2025 Oct 16;16:79. doi: 10.1186/s13293-025-00768-7 (PMC12532474; doi:10.1186/s13293-025-00768-7)

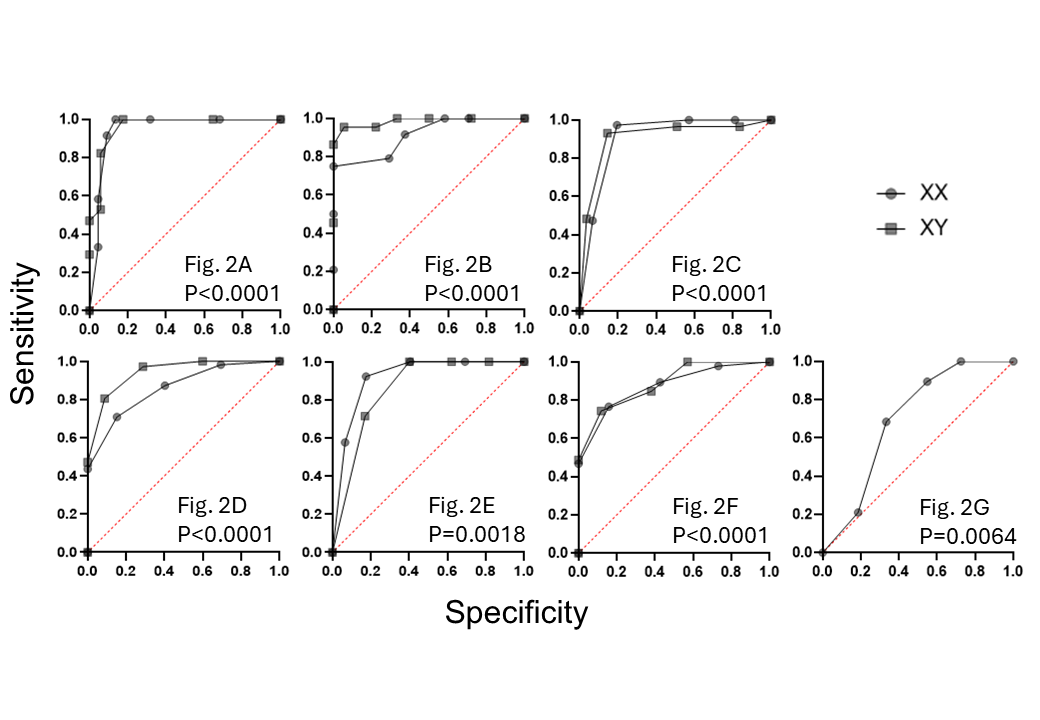

Supplement: Supplementary file 2 — Fig. S1: Receiver-operating characteristic (ROC) curves for the logistic regressions shown in Fig. 2. [file 13293_2025_768_MOESM2_ESM.png]
